# Supplementary material for: Incidence and Risk Factors for Development of Cardiac Toxicity in Adult Patients with Newly Diagnosed Acute Myeloid Leukemia
Source: Cancers (Basel). 2023 Apr 12;15(8):2267. doi: 10.3390/cancers15082267 (PMC10136564; doi:10.3390/cancers15082267)

Supplementary material: Incidence and risk factors for development of cardiac toxicity in adult patients with newly diagnosed acute myeloid leukemia

Supplementary Table S1. Baseline cardiac characteristics in all patients

Characteristic	Overall cohort	
	**Median (range)**	**Number of patients n=571 (%)**
Relevant cardiac comorbidity		571
Yes		82 (14)
No		489 (86)
All cardiac comorbidity		571
Yes		215 (38)
No		356 (62)
Baseline cardiac medication		571
Yes		265 (46)
No		306 (54)
Baseline cardiac medication	0 (0-9)	571
0		306 (54)
1-2		176 (31)

3-4		73 (13)
>4		16 (3)
Baseline electrocardiogram		571
Normal		278 (49)
Abnormal non clinically significant		128 (22)
Abnormal clinically significant		17 (3)
Not available		148 (26)
Baseline QTcF electrocardiogram	421 (347-556)	409
QTcF <450		359 (88)
QTcF 450-480		38 (9)
QTcF 481-500		7 (2)
QTcF >500		5 (1)
Baseline echocardiogram		571
Normal		242 (42)
Abnormal		70 (12)
Not available		259 (45)
Baseline ejection fraction	66 (35-92)	221
LVEF <50		6 (3)
LVEF ≥50		215 (97)

Heart failure at diagnosis		571
Yes		39 (7)
No		532 (93)
Baseline NYHA	0 (0-4)	571
0		532 (93)
1		12 (2)
2		11 (2)
3		13 (2)
4		3 (1)
Cardioischemic signs or symptoms at diagnosis		571
Yes		11 (2)
No		560 (98)

Supplementary Table S2. Demographic, clinical, and biological characteristics of AML patients at diagnosis according to therapeutic approach (Intensive vs HMA vs LDAC-based vs Trial vs BSC).

Characteristic	Intensive		HMA		LDAC-based		Intensive trial		Non intensive trial		BSC		P value
	Median (range)	n (%)	Median (range)	n (%)	Median (range)	n (%)	Median (range)	n (%)	Median (range)	n (%)	Median (range)	n (%)	

**N**		218 (46%)		7 (1%)		68 (13%)		67 (13)		165 (31%)		46 (9%)	
**Age, years**	54 (18-68)	218	69 (49-78)	7	71 (51-91)	68	63 (29-72)	67	74 (57-98)	165	77 (54-94)	46	<0.001
<65		210 (96)		3 (43)		7 (10)		40 (60)		4 (2)		8 (17)	<0.001
≥65-<75		8 (4)		1 (14)		35 (51)		27 (40)		79 (48)		10 (22)	
≥75		0		3 (43)		26 (38)		0		82 (50)		28 (61)	
**Gender**		218		7		68		67		165		46	
Male		121 (56)		5 (71)		42 (62)		40 (60)		97 (59)		26 (57)	0.897
Female		97 (45)		2 (29)		26 (38)		27 (40)		68 (41)		20 (43)	
**ECOG**	1 (0-4)	218	1 (0-2)	7	1 (0-4)	68	0 (0-3)	67	1 (0-4)	165	3 (0-4)	46	<0.001
0		101 (46)		3 (43)		16 (24)		35 (52)		47 (28)		2 (4)	<0.001
1		85 (39)		2 (29)		22 (32)		28 (42)		79 (48)		12 (26)	
2		24 (11)		2 (29)		17 (25)		3 (4)		28 (17)		7 (15)	
3		5 (2)		0		6 (10)		1 (1)		10 (6)		11 (24)	
4		3 (1)		0		7 (10)		0		1 (1)		14 (30)	
**ECOG Group**		218		7		68		67		165		46	
0-1		186 (85)		5 (71)		38 (56)		63 (94)		126 (76)		14 (30)	<0.001
≥2		32 (15)		2 (29)		31 (44)		4 (6)		39 (24)		32 (70)	
**Comorbidities**		218		7		68		67		165		46	
Yes		108 (50)		5 (71)		52 (76)		40 (60)		131 (79)		42 (91)	<0.001
No		110 (50)		2 (29)		16 (24)		27 (40)		34 (21)		4 (9)	
**Type of AML**		217		7		67		66		164		46	
De novo		158 (73)		1 (14)		31 (46)		44 (67)		94 (57)		20 (43)	<0.001
Secondary to MDS/MPS		25 (12)		6 (86)		21 (31)		14 (21)		48 (29)		18 (39)	
Therapy related		34 (16)		0		15 (22)		8 (12)		22 (13)		8 (17)	

**FAB subtype**		204		6		64		48		134		35	
M0		10 (5)		0		5 (8)		2 (4)		10 (7)		2 (6)	0.002
M1		35 (17)		0		6 (9)		16 (33)		14 (10)		1 (3)	
M2		42 (21)		0		11 (17)		4 (8)		27 (20)		4 (11)	
M4		36 (18)		0		9 (14)		8 (17)		23 (17)		7 (20)	
M5		28 (14)		0		7 (11)		6 (13)		10 (7)		4 (11)	
M6		10 (5)		1 (17)		2 (3)		1 (2)		8 (6)		0	
M7		2 (1)		0		0		0		2 (1)		1 (3)	
NA/other		41 (20)		5 (83)		24 (38)		11 (23)		40 (30)		16 (46)	
**WHO 2016**		218		7		68		67		164		46	
AML-RGA		91 (42)		0		14 (21)		23 (34)		24 (15)		2 (4)	<0.001
AML-NOS		29 (13)		0		8 (12)		5 (7)		23 (14)		3 (7)	
AML-MRC		62 (28)		6 (86)		30 (44)		26 (39)		86 (52)		30 (65)	
Myeloid sarcoma		1 (0.5)		0		0		0		1 (1)		0	
t-AML		18 (8)		0		13 (19)		5 (7)		11 (7)		5 (11)	
Ambiguous lineage		4 (2)		0		1 (1)		1 (1)		2 (1)		0	
BPDCN		0		0		0		0		2 (1)		0	
Not available		13 (6)		1 (14)		2 (3)		7 (10)		15 (9)		6 (13)	
**Extramedullary disease**		217		7		68		67		164		46	
Yes		48 (22)		0		9 (13)		11 (16)		27 (16)		5 (11)	0.213
No		169 (78)		7 (100)		59 (87)		56 (84)		137 (84)		41 (89)	
**WBC, ×10^9/L**	10 (0.4-434.3)	218	2.7 (0.5-75)	7	8.9 (0.4-317.9)	68	6.8 (0.3-246.4)	67	5.7 (0.6-223.6)	165	13.7 (0.3-202)	46	0.041
≤ 5		87 (40)		5 (71)		27 (40)		30 (45)		82 (50)		10 (22)	0.07

5-10		22 (10)		1 (14)		10 (15)		8 (12)		18 (11)		9 (20)	
10-50		62 (28)		0		15 (22)		14 (21)		45 (27)		17 (37)	
> 50		47 (22)		1 (14)		16 (24)		15 (22)		20 (12)		10 (22)	
**Neutrophil count, ×10^9/L**		204		5		66		52		142		38	
≤ 0.5		69 (34)		2 (40)		20 (30)		20 (38)		37 (26)		10 (26)	0.27
0.5-1		38 (19)		2 (40)		11 (17)		5 (10)		24 (17)		3 (8)	
> 1		97 (48)		1 (20)		35 (53)		27 (52)		81 (57)		25 (66)	
**Hemoglobin, g/dL**	8.8 (3.8-15.5)	218	8.8 (6.9-14.3)	7	8.9 (4.8-13.9)	68	9.1 (5.3-13.1)	67	8.6 (3.8-14.2)	165	8.6 (2.9-12.7)	46	0.53
≤ 10		159 (73)		5 (71)		52 (76)		49 (73)		134 (81)		35 (76)	0.55
> 10		59 (27)		2 (29)		16 (24)		18 (27)		31 (19)		11 (24)	
**Platelet count, ×10^9/L**	53 (2-1442)	218	54 (9-816)	7	42 (1-599)	68	73 (9-330)	67	54 (7-708)	165	32 (5-613)	45	0.004
≤ 20		34 (16)		2 (29)		21 (31)		7 (10)		30 (18)		15 (33)	0.004
> 20		184 (84)		5 (71)		47 (69)		60 (90)		135 (82)		30 (67)	
**PB blasts, %**		214		7		68		67		163		44	
≤ 50		146 (68)		6 (86)		49 (72)		46 (69)		137 (84)		33 (75)	0.016
> 50		68 (32)		1 (14)		19 (28)		21 (31)		26 (16)		11 (25)	
**BM blasts, %**	58 (0-100)	216	28 (21-90)	7	57 (8-100)	67	50 (18-99)	67	38 (1-100)	165	37 (20-99)	37	<0.001
≤ 30		47 (22)		4 (57)		16 (24)		17 (25)		60 (36)		14 (38)	0.021
>30-≤70		94 (44)		2 (29)		30 (45)		25 (37)		71 (43)		12 (32)	
> 70		75 (35)		1 (14)		21 (31)		25 (37)		34 (21)		11 (30)	
**Creatinine, mg/dL**	0.8 (0.1-6.9)	218	0.9 (0.6-3.6)	6	0.9 (0.4-3.2)	68	0.8 (0.4-7.4)	67	0.9 (0.4-3)	165	1.1 (0.3-5.4)	46	<0.001
≤ 1.3		201 (92)		5 (83)		55 (81)		59 (88)		135 (82)		27 (59)	<0.001

> 1.3		17 (8)		1 (17)		13 (19)		8 (12)		30 (18)		19 (41)	
**Urea, mg/dL**	30 (6-174)	208	33 (21-64)	6	41 (18-149)	66	32 (10-134)	67	41 (12-156)	163	60 (23-158)	44	<0.001
≤ 50		185 (89)		5 (83)		45 (68)		59 (88)		116 (71)		19 (43)	<0.001
> 50		23 (11)		1 (17)		21 (32)		8 (12)		47 (29)		25 (57)	
**Uric acid, mg/dL**	4.5 (1-14.6)	174	3.6 (1.7-7.6)	5	5.2 (2.3-17.1)	59	4.3 (2.3-29)	61	5.7 (1.3-15.8)	156	6.4 (1.4-15.7)	34	<0.001
≤ 7		152 (87)		4 (80)		45 (76)		55 (90)		121 (78)		21 (62)	0.003
> 7		22 (13)		1 (20)		14 (24)		6 (10)		35 (22)		13 (38)	
**Bilirubin, mg/dL**	0.6 (0.1-6.2)	212	0.7 (0.5-2.2)	5	0.7 (0.3-2.1)	64	0.6 (0.2-1.8)	67	0.7 (0.1-2.4)	164	0.8 (0.2-4.8)	41	<0.001
≤ 1.2		179 (84)		3 (60)		55 (86)		66 (99)		149 (91)		32 (78)	0.003
> 1.2		33 (16)		2 (40)		9 (14)		1 (1)		15 (9)		9 (22)	
**AST, U/L**	23 (6-781)	210	23 (14-42)	6	25 (8-88)	66	21 (10-229)	63	20 (7-112)	159	26 (7-1085)	45	0.036
≤ 50		181 (86)		6 (100)		57 (86)		58 (92)		147 (92)		33 (73)	0.008
> 50		29 (14)		0		9 (14)		5 (8)		12 (8)		12 (27)	
**ALT, U/L**	21 (3-714)	217	16 (8-37)	6	17 (5-120)	67	21 (7-306)	67	15 (3-146)	165	15 (6-593)	44	<0.001
≤ 50		185 (85)		6 (100)		59 (88)		56 (84)		158 (96)		40 (91)	0.017
> 50		32 (15)		0		8 (12)		11 (16)		7 (4)		4 (9)	
**AP, U/L**	72 (26-1158)	191	77 (52-126)	6	79 (39-472)	60	73 (27-542)	65	73 (30-416)	162	76 (39-326)	29	0.6
≤ 150		170 (89)		6 (100)		54 (90)		57 (88)		148 (91)		23 (79)	0.46
> 150		21 (11)		0		6 (10)		8 (12)		14 (9)		6 (21)	
**Albumin, g/dL**	3.7 (1.6-5.1)	196	3.5 (3-4.4)	5	3.5 (2-4.8)	58	3.8 (2.4-5.2)	65	3.9 (2.2-5.2)	160	3.6 (1.7-4.9)	34	<0.001

≤ 3.5		85 (43)		3 (60)		30 (52)		21 (32)		48 (30)		17 (50)	0.011
> 3.5		111 (57)		2 (40)		28 (48)		44 (68)		112 (70)		17 (50)	
**LDH, U/L**	618 (138-19670)	213	383 (152-1306)	6	637 (201-11380)	65	309 (101-1496)	66	426 (143-3845)	164	941 (149-42630)	44	<0.001
≤ 600		100 (47)		5 (83)		33 (51)		49 (74)		105 (64)		16 (36)	<0.001
> 600		103 (53)		1 (17)		32 (49)		17 (26)		59 (36)		28 (64)	
**Fibrinogen**	467 (34-1150)	215	485 (287-658)	4	454 (238-831)	64	537 (274-944)	64	504 (145-1002)	157	517 (93-867)	44	0.128
≤ 170		3 (1)		0		0		0		1 (1)		2 (5)	0.24
> 170		212 (99)		4 (100)		64 (100)		64 (100)		156 (99)		42 (95)	
**Dimer-D, ng/ml**	740 (39-128400)	136	394 (394-394)	1	959 (142-99000)	34	1632 (4-1828000)	62	1271 (11-523300)	114	3358 (22-79630)	20	0.001
≤ 500		50 (37)		0		7 (21)		7 (11)		23 (20)		3 (15)	<0.001
> 500		86 (63)		1 (100)		27 (79)		55 (89)		91 (80)		17 (85)	
**Prothrombin time**		186		5		63		62		144		41	
Prolonged		64 (34)		0		24 (38)		19 (31)		46 (32)		22 (54)	0.065
Normal		122 (66)		5 (100)		39 (62)		43 (69)		98 (68)		19 (46)	
**APTT**		211		6		66		64		150		44	
Prolonged		41 (19)		2 (33)		11 (17)		2 (3)		12 (8)		5 (11)	0.002
Normal		170 (81)		4 (67)		55 (83)		62 (97)		138 (92)		39 (89)	
**Cytogenetics**		218		7		68		67		165		46	
Normal		82 (38)		4 (57)		35 (51)		26 (39)		60 (36)		5 (11)	<0.001
Abnormal		115 (53)		1 (14)		28 (41)		37 (55)		85 (52)		26 (57)	
No metaphases		18 (8)		2 (29)		4 (6)		4 (6)		15 (9)		4 (9)	

Not available		3 (1)		0		1 (1)		0		5 (3)		11 (24)	
**MRC Cytogenetic risk**		201		5		66		63		147		35	
Favorable		23 (11)		0		1 (2)		2 (3)		1 (1)		0	<0.001
Intermediate		119 (59)		4 (80)		45 (69)		41 (65)		86 (59)		12 (34)	
Adverse		59 (29)		1 (20)		20 (30)		20 (32)		60 (41)		23 (66)	
**FLT3-ITD**		200		3		56		67		159		32	
0-0.06		168 (84)		3 (100)		51 (91)		50 (75)		149 (94)		31 (97)	0.016
0.06-0.5		9 (5)		0		3 (5)		7 (10)		4 (3)		1 (3)	
0.5-0.8		10 (5)		0		1 (2)		7 (10)		5 (3)		0	
>0.8		13 (7)		0		1 (2)		3 (4)		1 (1)		0	
**FLT3-TKD**		199		3		56		66		158		32	
Positive		11 (6)		0		2 (4)		5 (8)		5 (3)		3 (9)	0.058
Negative		186 (93)		3 (100)		52 (93)		60 (91)		152 (96)		26 (81)	
Not available		2 (1)		0		2 (4)		1 (2)		1 (1)		3 (9)	
**NPM1**		201		3		56		67		156		31	
Positive		66 (33)		0		12 (21)		19 (28)		21 (13)		2 (6)	<0.001
Negative		135 (67)		3 (100)		44 (79)		48 (72)		135 (87)		29 (94)	
**CEBPA**		218		7		68		67		165		46	
Positive		6 (3)		0		2 (3)		1 (1)		5 (3)		0	<0.001
Negative		98 (45)		1 (14)		27 (40)		51 (76)		122 (74)		18 (39)	
Not available		114 (52)		6 (86)		39 (57)		15 (23)		38 (23)		28 (61)	
**IDH**		218		7		68		67		165		46	
IDH1 positive		5 (2)		0		1 (1)		7 (10)		9 (5)		1 (2)	<0.001
IDH2 positive		19 (9)		0		4 (6)		9 (13)		20 (12)		2 (4)	
Negative		111 (51)		1 (14)		20 (29)		39 (58)		83 (50)		12 (26)	
Not available		83 (38)		6 (86)		43 (63)		12 (18)		53 (32)		31 (67)	

**KIT**		218		7		68		64		164		46	
Positive		6 (3)		0		0		0		1 (1)		0	<0.001
Negative		38 (17)		1 (14)		8 (12)		47 (73)		60 (37)		3 (7)	
Not available		174 (80)		6 (86)		60 (88)		17 (27)		103 (63)		43 (93)	
**Relevant Cardiac comorbidity**		218		7		68		67		165		46	
Yes		19 (9)		2 (29)		11 (16)		9 (13)		32 (19)		9 (20)	0.043
No		199 (91)		5 (71)		57 (84)		58 (87)		133 (81)		37 (80)	
**Previous anthracycline**		218		7		68		67		165		46	
Yes		5 (2)		1 (14)		6 (9)		4 (6)		4 (2)		7 (15)	0.001
No		213 (98)		6 (86)		62 (91)		63 (94)		161 (98)		39 (85)	

Supplementary Table S3. Overall cohort: Multivariate analyses of prognostic factors for development of fatal cardiac events

Covariate	Unfavorable category	Univariate Analysis	Multivariate Analysis	P value
		P value	HR (95% CI)	
Age	≥65	0.247	1.1 (0.3-4.7)	0.88
ECOG	>2	0.56	1.3 (0.4-4)	0.66
Relevant cardiac antecedents	Yes	<0.001	1.9 (2.8-16.6)	<0.001
Front-line therapy	Intensive	0.37	2 (0.4-9.4)	0.4
Front-line clinical trial	Yes	0.062	2.4 (0.1-1.2)	0.095
Use of FLT3 inhibitors	Yes	0.757	2.3 (0.1-2.9)	0.38

Abbreviations: HR: hazard ratio; CI: confidence interval

Supplementary Table S4. Overall cohort: Multivariate analyses of prognostic factors for development of non-fatal cardiac events

Covariate	Unfavorable category	Univariate Analysis	Multivariate Analysis	P value
		P value	HR (95% CI)	
Age	≥65	<0.001	2.2 (1.5-3.3)	<0.001
ECOG	>2	0.626	1.1 (0.8-1.5)	0.54
Relevant cardiac antecedents	Yes	0.004	1.4 (1.1-2)	0.023
Front-line therapy	Non-intensive	0.17	1.8 (1.2-2.8)	0.004
Front-line clinical trial	Yes	<0.001	1.2 (0.6-1.1)	0.21
Use of FLT3 inhibitors	Yes	0.28	1.0 (0.6-1.7)	0.89

Abbreviations: HR: hazard ratio; CI: confidence interval

Supplementary Table S5. 1L study cohort (from index date to last follow-up): First line Intensive chemotherapy patients only. Crude incidence of first cardiac events (Grade 1-2 vs grade 3-4 vs grade 5 vs no cardiac event) according to time of occurrence (before starting therapy, during induction cycle, consolidation, after stem cell transplant, off therapy/follow-up)

Characteristic	N	No cardiac event	Grade 1-2	Grade 3-4	Grade 5
		n (%)	n (%)	n (%)	n (%)
**N**	285	165 (57.9)	40 (14)	76 (26.7)	4 (1.4)
**Before starting therapy**	285	276 (96.8)	1 (0.4)	8 (2.8)	0 (0)
**Induction**	285	222 (77.9)	20 (7)	42 (14.7)	1 (0.4)
**Consolidation**	169	142 (84)	11 (6.5)	14 (8.3)	2 (1.2)
**Allogeneic HSCT**	65	58 (89.2)	3 (4.6)	4 (6.2)	0 (0)
**Off therapy/ follow-up**	163	149 (91.4)	5 (3.1)	8 (4.9)	1 (0.6)

Supplementary Table S6. Intensive chemotherapy patients only: Crude and cumulative incidence of cardiac events (Fatal vs non-fatal vs no cardiac event) according to demographic, clinical, and biological characteristics of intensive chemotherapy patients at diagnosis.

	No cardiac event	P value*	Fatal				Non-fatal			
Characteristic	N (%)		Crude incidence N (%)	Cumulative incidence			Crude incidence N (%)	Cumulative incidence		
				At 6 months, %	At Last FU,%	P value		At 6 months, %	At Last FU, %	P value
**N**	125 (43.9)		10 (3.5)	1.9	6.3		150 (52.6)	42.5	54.7	
**Age**	125		10				150			
**<65 years**	117 (46.8)	0.022	9 (3.6)	1.7	6.2	0.79	124 (49.6)	38.7	51.5	<0.001
**≥65 years**	8 (22.9)		1 (2.9)	3.6	3.6		26 (74.3)	69.4	75.6	
**Relevant cardiologic antecedents**	125		10				150			
**No**	120 (46.7)	<0.001	6 (2.3)	1.3	5	<0.001	131 (51)	40.3	52.4	0.02
**Yes**	5 (17.9)		4 (14.3)	7.4	18.5		19 (67.9)	64.4	74.4	
**Previous anthracycline treatment**	125		10				150			
**No**	120 (44.8)	0.11	8 (3)	1.2	5.8	0.008	140 (52.2)	42.1	54.1	0.27
**Yes**	5 (29.4)		2 (11.8)	13.4	13.4		10 (58.8)	50.4	57.8	
**ECOG at diagnosis**	125		10				150			
**<2**	109 (43.8)	0.76	8 (3.2)	1.7	6.1	0.34	132 (53)	42.9	55.1	0.79
**≥2**	16 (44.4)		2 (5.6)	2.8	6.8		18 (50)	39.8	52.6	
**FLT3-ITD status**	117		10				140			

Negative	91 (42.5)	0.56	9 (4.2)	2.5	7.5	0.46	114 (53.3)	43.6	55.8	0.66
Positive	26 (49.1)		1 (1.9)	0	2.3		26 (49.1)	39.6	49.3	
**Inclusion in clinical trial**	125		10				150			
No	103 (47.3)	0.11	7 (3.2)	1.5	5.8	0.36	108 (49.5)	37.9	51.1	0.011
Yes	22 (32.8)		3 (4.5)	3.2	5.2		42 (62.7)	57.7	64.2	
**Use of FLT3 inhibitors**	125		10				150			
No	111 (44.8)	0.67	9 (3.6)	2.2	3.9	0.89	128 (51.6)	40.8	53.4	0.20
Yes	14 (37.8)		1 (2.7)	0	3.1		22 (59.5)	54.1	59.7	

*This P value compares the crude incidence between the 3 groups

Supplementary Table S7. Non-intensive chemotherapy patients only: Crude and cumulative incidence of cardiac events (Fatal vs non-fatal vs no cardiac event) according to demographic, clinical, and biological characteristics of intensive chemotherapy patients at diagnosis.

Characteristic	No cardiac event	P value*	Fatal				Non-fatal			
	N (%)		Crude Incidence N (%)	Cumulative incidence			Crude Incidence N (%)	Cumulative incidence		
				At 6 months, %	At Last FU, %	P value		At 6 months, %	At Last FU, %	P value
**N**	93 (38.8)		9 (3.8)	2.2	6.8		138 (57.5)	45.2	59.6	
**Age**	93		9				138			
<65 years	11 (78.6)	0.007	0	0	0	0.54	3 (21.4)	21.4	21.4	0.059
≥65 years	82 (36.3)		9 (4)	2.4	7.2		135 (59.7)	46.6	62	

**Relevant cardiologic antecedents**	93		9				138			
**No**	82 (42.1)	0.003	4 (2.1)	1.1	3.7	0.002	109 (55.9)	43.8	57.3	0.1
**Yes**	11 (24.4)		5 (11.1)	7.1	22.1		29 (64.4)	51.4	70.6	
**Previous anthracycline treatment**	93		9				138			
**No**	87 (38.8)	0.7	9 (4)	2.4	7.2	0.42	128 (57.1)	44.8	59.1	0.94
**Yes**	6 (37.5)		0	0	0		10 (62.5)	50	56.3	
**ECOG**	93		9				138			
**<2**	64 (37.9)	0.83	7 (4.1)	2.4	7.4	0.84	98 (58)	43.9	60.3	0.66
**≥2**	29 (40.9)		2 (2.8)	1.5	4.8		40 (56.3)	48.3	57.6	
**FLT3-ITD status**	80		9				129			
**Negative**	70 (36.1)	0.86	8 (4.1)	2.2	7.5	0.82	116 (59.8)	44.9	62.1	0.94
**Positive**	10 (41.7)		1 (4.2)	4.8	4.8		13 (54.2)	51.3	55.8	
**Inclusion in clinical trial**	93		9				138			
**No**	37 (49.3)	0.047	1 (1.3)	0	8	0.24	37 (49.3)	36	48	0.071
**Yes**	56 (33.9)		8 (4.9)	3.2	7.1		101 (61.2)	49.4	63.9	
**Use of FLT3 inhibitors**	93		9				138			
**No**	93 (38.9)	0.69	9 (3.8)	2.2	6.8	0.86	137 (57.3)	44.9	59.5	NA
**Yes**	0		0	0	0		1 (100)	NA	NA	

*This P value compares the crude incidence between the 3 groups

Supplementary Table S8. Overall study cohort: Crude and cumulative incidence of QTcF prolongation events (grade 0 vs grade 1-2 vs grade 3-4 vs grade 5) according to demographic, clinical, and biological characteristics of patients at diagnosis.

	No QTcF prolongation	P value*	Grade 1-2 QTcF prolongation				Grade 3-4 QTcF prolongation**			
Characteristic	N (%)		Crude incidence N (%)	Cumulative incidence			Crude incidence N (%)	Cumulative incidence		
				At 6 months, %	At Last FU,%	P value		At 6 months, %	At Last FU,%	P value
**N**	452 (86)		59 (11.2)	8.4	11.6		14 (2.7)	1.5	3.1	
**Age**	452		59				14			
**<65 years**	234 (88.6)	0.01	20 (7.6)	6.1	7.6	0.007	10 (3.8)	1.5	4.3	0.16
**≥65 years**	218 (83.5)		39 (14.9)	10.8	16		4 (1.5)	1.5	1.5	
**Relevant cardiologic antecedents**	452		59				14			
**No**	397 (87.8)	0.015	44 (9.7)	7.3	10.2	0.008	11 (2.4)	1.8	2.5	0.38
**Yes**	55 (75.3)		15 (20.6)	15.1	20.9		3 (4.1)	0	6	
**Previous anthracycline treatment**	452		59				14			
**No**	425 (86.4)	0.75	54 (11)	8.1	11.2	0.49	13 (2.6)	1.4	3.1	0.87
**Yes**	27 (81.8)		5 (15.2)	12.1	12.1		1 (3)	3.3	3.3	
**ECOG at diagnosis**	452		59				14			
**<2**	356 (85.2)	0.48	50 (12)	8.9	12.4	0.29	12 (2.9)	1.4	3.4	0.56
**≥2**	96 (89.7)		9 (8.4)	6.6	8.6		2 (1.9)	1.9	1.9	
**FLT3-ITD status**	416		57				12			
**Negative**	347 (85.1)	0.5	51 (12.5)	9.1	13	0.26	10 (2.5)	1.2	3	0.96
**Positive**	69 (89.6)		6 (7.8)	6.5	7.9		2 (2.6)	1.3	2.7	

**Treatment chemotherapy**	452		59				14			
**Intensive**	245 (86)	0.38	30 (10.5)	8.4	10.6	0.59	10 (3.5)	1.4	4.1	0.24
**Non-intensive**	207 (86.3)		29 (12.1)	8.4	12.9		4 (1.7)	1.7	1.7	
**Inclusion in clinical trial**	452		59				14			
**No**	268 (91.5)	<0.001	17 (5.8)	4.4	5.8	<0.001	8 (2.7)	1	2.6	0.87
**Yes**	184 (79.3)		42 (18.1)	13.4	20.5		6 (2.6)	2.2	3.1	
**Use of FLT3 inhibitors**	452		59				14			
**No**	424 (87.1)	0.041	50 (10.3)	7.6	10.6	0.008	13 (2.7)	1.4	2.6	0.95
**Yes**	28 (73.7)		9 (23.7)	18.4	24.4		1 (2.6)	2.6	2.6	

*This P value compares the crude incidence between the 3 groups; ** All were grade 3

Supplementary Table S9. Overall cohort: Crude and cumulative incidence of arrhythmia (grade 0 vs grade 1-2 vs grade 3-4 vs grade 5) according to demographic, clinical, and biological characteristics of patients at diagnosis.

	No arrhythmia	P value*	Grade 1-2 arrhythmia				Grade 3-4 arrhythmia				Grade 5 arrhythmia			
**Characteristic**	**N (%)**		**Crude incidence N (%)**	**Cumulative incidence**			**Crude incidence N (%)**	**Cumulative incidence**			**Crude incidence N (%)**	**Cumulative incidence**		
				**At 6 months, %**	**At Last FU,%**	**P value**		**At 6 months , %**	**At Last FU,%**	**P**		**At 6 months, %**	**At Last FU,%**	**P value**
**N**	461 (89)		10 (1.9)	1.2	2.3		48 (9.1)	7.3	9.2		6	0.4	1	
**Age**	461		10				48				6			
**<65 years**	242 (91.7)	0.045	3 (1.1)	0.4	1.7	0.15	16 (6.1)	4.9	6.1	0.013	3 (1.1)	0.8	2.3	0.61
**≥65 years**	219 (83.9)		7 (2.7)	2	2.9		32 (12.3)	9.6	12.4		3 (1.1)	0.8	1.2	

**Relevant cardiologic antecedents**	461		10				48							
**No**	402 (88.9)	0.12	9 (2)	1.3	2.1	0.69	37 (8.2)	6.2	8.2	0.055	4 (0.9)	0.5	1.8	0.14
**Yes**	59 (80.8)		1 (1.4)	0	1.9		11 (15.1)	13.7	15.1		2 (2.7)	2.8	2.8	
**Previous anthracycline treatment**	461		10				48							
**No**	433 (88)	0.71	9 (1.8)	1.2	2.2	0.63	45 (9.2)	7.1	9.2	0.96	5 (1)	0.6	1.8	0.21
**Yes**	28 (84.8)		1 (3)	0	3.4		3 (9.1)	9.1	9.1		1 (3)	3.0	3.0	
**ECOG at diagnosis**	461		10				48							
**<2**	375 (89.7)	0.043	7 (1.7)	1.2	2.1	0.39	31 (7.4)	5.3	7.5	0.005	5 (1.2)	0.7	2.1	0.93
**≥2**	86 (80.4)		3 (2.8)	1	3.4		17 (15.9)	15	16		1 (0.9)	1	1	
**FLT3-ITD status**	425		8				46							
**Negative**	358 (87.7)	0.63	6 (1.5)	1.3	1.9	0.47	38 (9.3)	7.4	9.3	0.77	6 (1.5)	1.0	2.4	0.3
**Positive**	67 (87)		2 (2.6)	1	2.8		8 (10.4)	7.8	10.6		0	0	0	
**Treatment chemotherapy**	461		10				48							
**Intensive**	259 (90.9)	0.10	3 (1.1)	0.4	1.6	0.1	20 (7)	5.6	7	0.062	3 (1)	0.7	2.3	0.49
**Non-intensive**	202 (84.2)		7 (2.9)	2.1	3.1		28 (11.7)	9.2	11.8		3 (1.2)	0.9	1.3	
**Inclusion in clinical trial**	461		10				48							
**No**	261 (89.1)	0.40	3 (1)	0.3	1.4	0.067	26 (8.9)	7.9	8.9	0.82	3 (1)	0.7	1.9	0.54
**Yes**	200 (86.2)		7 (3)	2.2	3.2		22 (9.5)	6.5	9.6		3 (1.3)	0.9	1.3	
**Use of FLT3 inhibitors**	461		10				48							
**No**	425 (87.3)	0.54	10 (2.1)	1.3	1.9	0.39	46 (9.5)	7.6	9.5	0.38	6 (1.2)	0.8	1.9	0.52

Yes	36 (94.7)		0	0	0		2 (5.3)	2.6	5.3		0	0	0	

*This P value compares the crude incidence between the 4 groups

Supplementary Table S10. Overall study cohort: Crude and cumulative incidence of heart failure and related events (grade 0 vs grade 1-2 vs grade 3-4 vs grade 5) according to demographic, clinical, and biological characteristics of patients at diagnosis.

	No cardiac failure event	P value*	Grade 1-2 cardiac failure event				Grade 3-4 cardiac failure event				Grade 5 cardiac failure event			
Characteristic	N (%)		Crude incidence N (%)	Cumulative incidence			Crude incidence N (%)	Cumulative incidence			Crude incidence N (%)	Cumulative incidence		
				At 6 months, %	At Last FU, %	P value		At 6 months, %	At Last FU,%	P value		At 6 months , %	At Last FU, %	P value
**N**	428 (81.5)		7 (1.3)	1	1.4		79 (15)	12.2	15.3		11 (2.1)	1	2.4	
**Age**	428		7				79				11			
<65 years	217 (82.2)	0.23	6 (2.3)	1.5	2.4	0.071	36 (13.6)	11	13.9	0.32	5 (1.9)	0.4	2.2	0.58
≥65 years	211 (80.8)		1 (0.4)	0.4	0.4		43 (16.5)	13.5	16.7		6 (2.3)	1.6	2.8	
**Relevant cardiologic antecedents**	428		7				79				11			
No	376 (83.2)	0.008	6 (1.3)	0.9	1.4	0.93	64 (14.2)	11.3	14.4	0.13	6 (1.3)	0.7	1.6	0.001
Yes	52 (71.2)		1 (1.4)	1.4	1.4		15 (20.6)	18.1	21		5 (6.9)	2.8	8	
**Previous anthracycline treatment**	428		7				79				11			
No	403 (81.9)	0.65	7 (1.4)	1	1.5	0.5	72 (14.6)	12	14.9	0.34	10 (2)	0.8	2.4	0.57
Yes	25 (75.8)		0	0	0		7 (21.2)	15.4	21.7		1 (3)	3.1	3.1	
**ECOG at diagnosis**	428		7				79				11			

**<2**	349 (83.5)	0.06	6 (1.4)	1	1.5	0.76	54 (12.9)	10.1	13.2	0.005	9 (2.2)	1.2	2.5	0.99
**≥2**	79 (73.8)		1 (0.9)	0.9	0.9		25 (23.4)	20.6	23.6		2 (1.9)	0	2.7	
**FLT3-ITD status**	395		7				72				11			
**Negative**	334 (81.9)	0.81	5 (1.2)	1	1.3	0.36	60 (14.7)	11.8	14.9	0.79	9 (2.2)	1	2.6	0.79
**Positive**	61 (79.2)		2 (2.6)	1.3	2.8		12 (15.6)	13	15.7		2 (2.6)	1.3	2.8	
**Treatment chemotherapy**	428		7				79				11			
**Intensive**	230 (80.7)	0.42	6 (2.1)	1.4	2.2	0.1	43 (15.1)	12.3	15.4	0.99	6 (2.1)	0.7	2.5	0.93
**Non-intensive**	198 (82.5)		1 (0.4)	0.4	0.4		36 (15)	12.2	15.2		5 (2.1)	1.3	2.4	
**Inclusion in clinical trial**	428		7				79				11			
**No**	238 (81.2)	0.23	6 (2.1)	1.4	2.1	0.12	45 (15.4)	12.3	15.5	0.93	4 (1.4)	0	1.7	0.12
**Yes**	190 (81.9)		1 (0.4)	0.5	0.5		34 (14.7)	12.2	14.9		7 (3)	2.2	3.2	
**Use of FLT3 inhibitors**	428		7				79				11			
**No**	398 (81.7)	0.18	5 (1)	0.8	1.1	0.15	74 (15.2)	12.4	15.4	0.81	10 (2.1)	1.1	2.4	0.74
**Yes**	30 (79)		2 (5.3)	2.6	5.6		5 (13.2)	10.5	13.3		1 (2.6)	0	3	

*This P value compares the crude incidence between the 4 groups

Supplementary Table S11. Overall study cohort: Crude and cumulative incidence of cardioischemic events (grade 0 vs grade 1-2 vs grade 3-4 vs grade 5*) according to demographic, clinical, and biological characteristics of patients at diagnosis.

	No cardioischemic event	P value*	Grade 1-2 cardioischemic event	Grade 3-4 cardioischemic event

Characteristic	N (%)		Crude incidence N (%)	Cumulative incidence			Crude incidence N (%)	Cumulative incidence		
				At 6 months, %	At Last FU,%	P value		At 6 months, %	At Last FU,%	P value
**N**	505 (96.2)		11 (2.1)	1.5	2.1		9 (1.7)	1.9	4.1	
**Age**	505		11				9			
**<65 years**	255 (96.6)	0.62	4 (1.5)	1.1	1.6	0.35	5 (1.9)	1.5	3.8	0.52
**≥65 years**	250 (95.8)		7 (2.7)	1.9	2.7		4 (1.5)	2.3	4.3	
**Relevant cardiologic antecedents**	505		11				9			
**No**	436 (96.5)	0.7	9 (2)	1.3	2	0.69	7 (1.6)	1.5	3.9	0.48
**Yes**	69 (94.5)		2 (2.7)	2.7	2.7		2 (2.7)	4.1	5.5	
**Previous anthracycline treatment**	505		11				9			
**No**	474 (96.3)	0.77	10 (2)	1.4	2.1	0.69	8 (1.6)	1.8	4	0.48
**Yes**	31 (93.9)		1 (3)	3.1	3.1		1 (3)	3	6.1	
**ECOG at diagnosis**	505		11				9			
**<2**	400 (95.7)	0.5	10 (2.4)	1.9	2.4	0.34	8 (1.9)	2.2	4.7	0.21
**≥2**	105 (98.1)		1 (0.9)	0	1		1 (0.9)	0.9	1.9	
**FLT3-ITD status**	468		10				7			
**Negative**	392 (96.1)	0.38	10 (2.5)	2	2.5	0.17	6 (1.5)	2	4.2	0.28
**Positive**	76 (98.7)		0	0	0		1 (1.3)	1.3	1.3	
**Treatment chemotherapy**	505		11				9			

**Intensive**	274 (96.1)	0.99	6 (2.1)	1.4	2.2	0.99	5 (1.8)	1.4	4.3	0.98
**Non-intensive**	231 (96.3)		5 (2.1)	1.7	2.1		4 (1.7)	2.5	3.8	
**Inclusion in clinical trial**	505		11				9			
**No**	279 (95.2)	0.35	7 (2.4)	1.4	2.4	0.62	7 (2.4)	1.7	5	0.31
**Yes**	226 (97.4)		4 (1.7)	1.7	1.7		2 (0.9)	2.2	2.8	
**Use of FLT3 inhibitors**	505		11				9			
**No**	468 (96.1)	0.68	10 (2.1)	1.4	2.1	0.76	9 (1.9)	1.8	4.1	0.84
**Yes**	37 (97.4)		1 (2.6)	2.6	2.6		0	2.6	2.6	

*This P value compares the crude incidence between the 3 groups

Supplementary Table S12. 1L cohort (from index date to first refractory/relapse): Crude and cumulative incidence of cardiac events (Fatal vs Non-fatal vs No cardiac event) according to demographic, clinical, and biological characteristics of patients at diagnosis

	**No cardiac event**	**P value***	**Fatal cardiac event**				**Non-fatal cardiac event**			
**Characteristic**	**N (%)**		**Crude incidence N (%)**	**Cumulative incidence**			**Crude incidence N (%)**	**Cumulative incidence**		
				**At 6 months, %**	**At Last FU, %**	**P value**		**At 6 months, %**	**At Last FU, %**	**P value**
**N**	296 (56.4)		7 (1.3)	1.2	2.8		222 (42.3)	37.8	43.3	
**Age**	296		7				222			

**<65 years**	165 (62.5)	0.013	4 (1.5)	1.2	3.1	0.92	95 (36)	31.3	37.2	0.001
**≥65 years**	131 (50.2)		3 (1.2)	1.2	1.2		127 (48.7)	44.4	49.4	
**Relevant cardiologic antecedents**	296		7				222			
**No**	270 (59.7)	<0.001	2 (0.4)	0.3	1.9	<0.001	180 (39.8)	35.6	40.5	0.002
**Yes**	26 (35.6)		5 (6.9)	7.4	7.4		42 (57.5)	51.8	61	
**All cardiologic antecedents**	296		7				222			
**No**	215 (65.6)	<0.001	2 (0.6)	0.4	1.9	0.02	111 (33.8)	30.2	34.6	<0.001
**Yes**	81 (41.1)		5 (2.5)	2.7	2.7		111 (56.4)	50.6	58.4	
**Previous anthracycline treatment**	296		7				222			
**No**	278 (56.5)	0.05	5 (1)	0.8	2.4	0.005	209 (42.5)	37.8	43.3	0.91
**Yes**	18 (54.6)		2 (6.1)	7.9	7.9		13 (39.4)	37.4	37.4	
**ECOG at diagnosis**	296		7				222			
**<2**	240 (57.4)	0.59	5 (1.2)	1	2.7	0.4	173 (41.4)	36.3	42.6	0.17
**≥2**	56 (52.3)		2 (1.9)	1.9	1.9		49 (45.8)	43.5	46.4	
**FLT3-ITD status**	269		7				209			
**Negative**	222 (54.4)	0.33	7 (1.7)	1.6	3.5	0.25	179 (43.9)	38.6	45.2	0.55
**Positive**	47 (61)		0	0	0		30 (39)	37.7	39	
**Treatment chemotherapy**	296		7				222			
**Intensive**	166 (58.3)	0.62	4 (1.4)	1.2	3.1	0.8	115 (40.4)	36	41.6	0.24
**Non-intensive**	130 (54.2)		3 (1.3)	1.3	1.3		107 (44.6)	39.9	45.1	
**Inclusion in clinical trial**	296		7				222			
**No**	185 (63.1)	0.002	3 (1)	0.7	2.4	0.28	105 (35.8)	31.5	36.6	<0.001

**Yes**	111 (47.8)		4 (1.7)	1.9	1.9		117 (50.4)	45.7	51.8	
**Use of FLT3 inhibitors**	296		7				222			
**No**	277 (56.9)	0.49	7 (1.4)	1.3	2.8	0.49	203 (41.7)	37	42.6	0.25
**Yes**	19 (50)		0	0	0		19 (50)	47.4	50	

*This P value compares the crude incidence between the 3 groups

Supplementary Table S13. 1L cohort: Multivariate analyses of prognostic factors for development of non-fatal cardiac events

Covariate	Unfavorable category	Univariate Analysis	Multivariate Analysis	
		P value	HR (95% CI)	P value
**Age**	**≥65**	0.001	1.8 (1.2-2.9)	0.007
**ECOG**	**>2**	0.17	1.3 (1-1.9)	0.08
**Relevant cardiologic antecedents**	**Yes**	<0.001	1.9 (1.4-2.6)	<0.001
**Front-line treatment**	**Non-intensive**	0.24	1.9 (1.2-3)	0.005
**Clinical trial**	**Yes**	<0.001	1.2 (0.6-1.2)	0.41
**Use of FLT3 inhibitors**	**Yes**	0.25	1.3 (0.7-2.2)	0.37

Abbreviations: HR: hazard ratio; CI: confidence interval

Supplementary Figure S1. Distribution of age at baseline AML diagnosis (diagnosed patients from HULaFe; n=571).

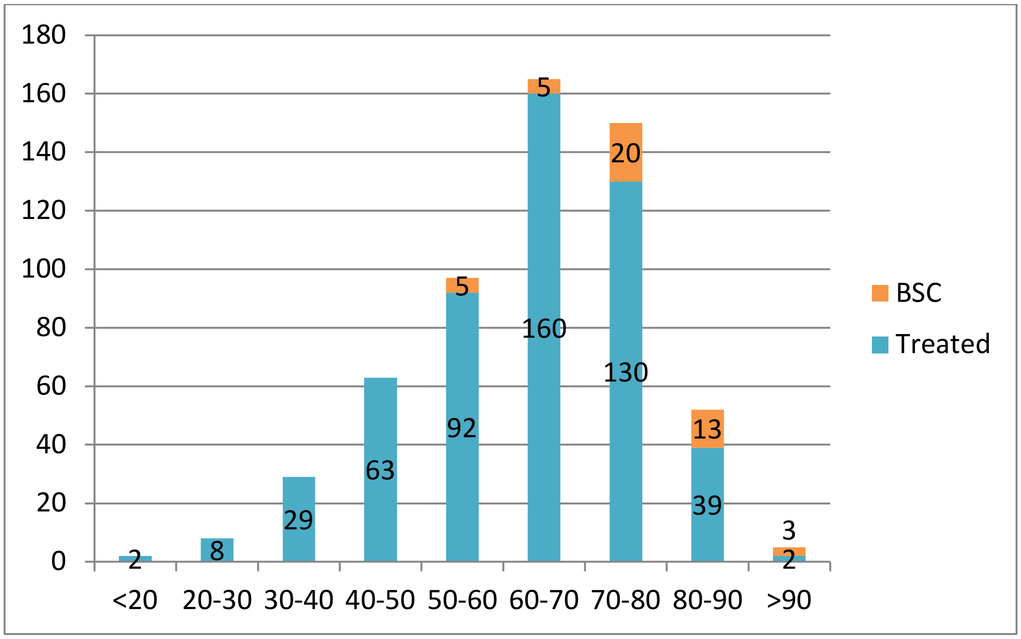

Supplementary Figure S2. Timing of life-threatening (requiring intensive management) cardiac events in the overall cohort.

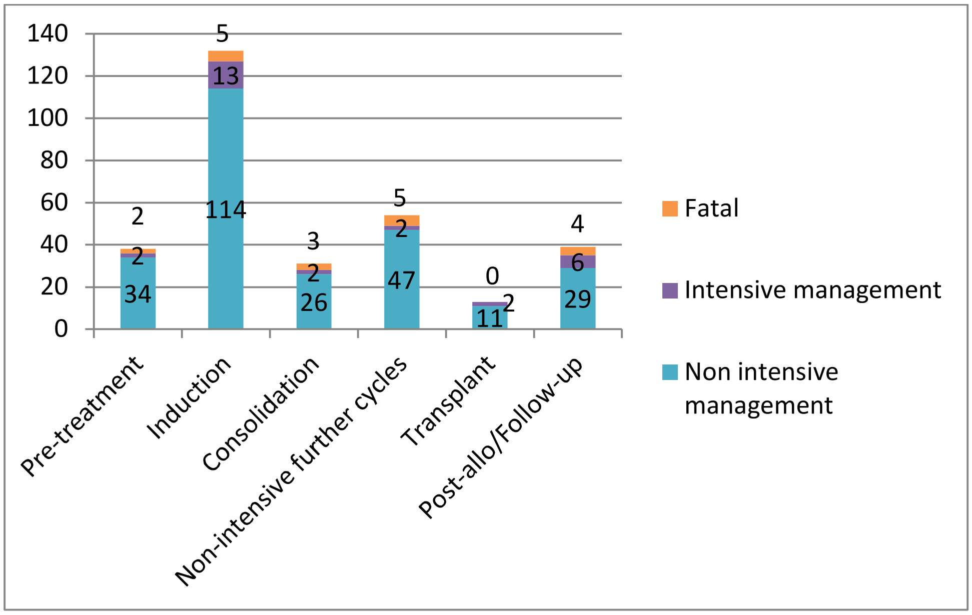

Supplementary Figure S3. Timing of QTcF prolongation events in the overall cohort.

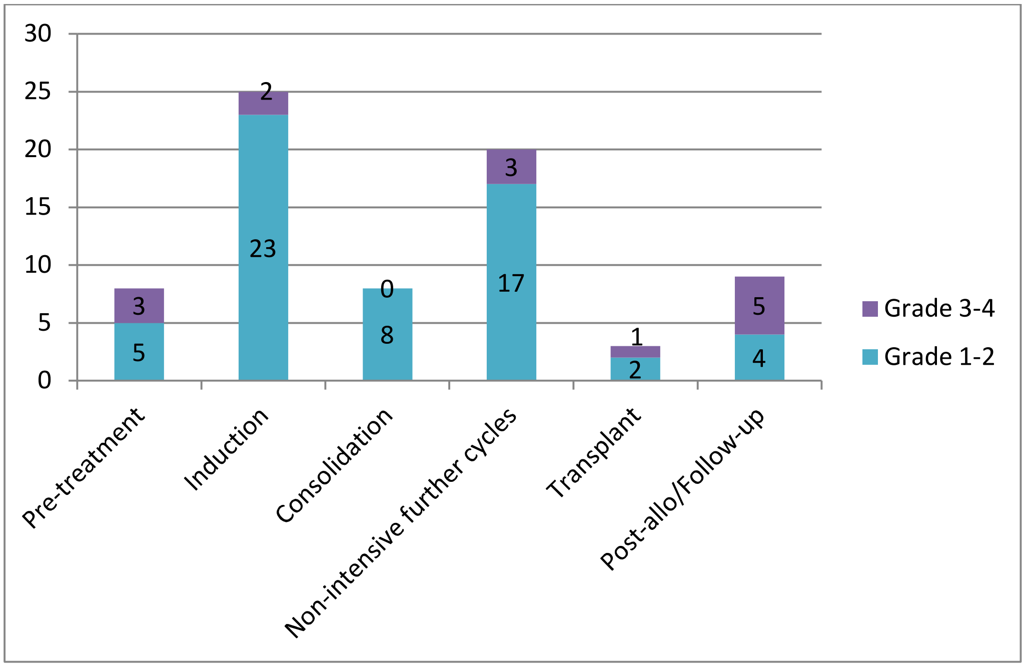

Supplementary Figure S4. Timing of arrhythmia events in the overall cohort.

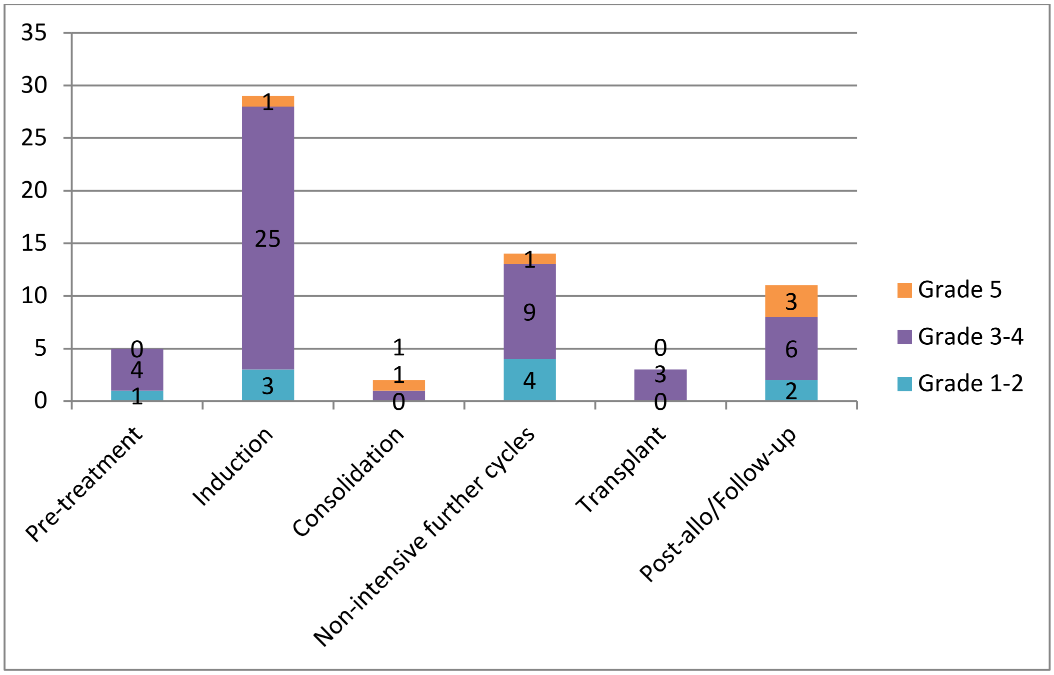

Supplementary Figure S5. Timing of heart failure events in the overall cohort.

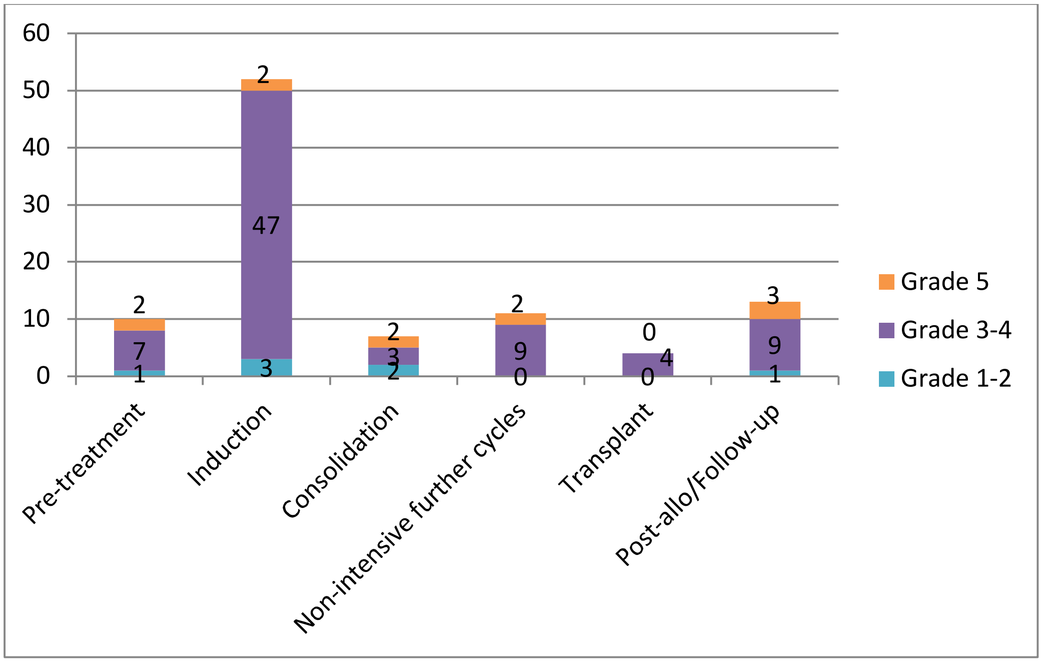

Supplement: Supplementary file 1 [file cancers-15-02267-s001.zip › cancers-2253885-SI.pdf]
